# Topiramate-induced psychosis in two members of the one family: a case report

**DOI:** 10.1186/1752-1947-2-195

**Published:** 2008-06-06

**Authors:** Ricardo Jorge Paixão José, Alastair Cairns, Christopher Babbs

**Affiliations:** 1 Department of Medicine, Salford Royal NHS Foundation Trust, Stott Lane, Salford, M6 8HD, UK

## Abstract

**Introduction:**

The use of topiramate has increased in recent years. It is now used by various specialties to treat a wide range of medical conditions. A small number of case reports describe psychosis as an adverse event of topiramate but most are in patients being treated for epilepsy and none describe a family history of this side effect.

**Case presentation:**

We present a case report of topiramate-induced psychosis in a patient with familial essential tremor, whose sister also developed this same adverse effect.

**Conclusion:**

Physicians need to be aware of psychosis as a rare but debilitating side effect of this drug. Several predictors have been identified and a careful history, including family history, needs to be obtained prior to initiation of therapy to stratify the risk.

## Introduction

Topiramate is a relatively new anti-epileptic drug. There is increasing interest in its use in a wide range of applications and therefore its use and side effects have great importance to general and specialist physicians. It is licensed in the United Kingdom for the management of epilepsy and migraine prophylaxis and is used 'off-label' in the management of essential tremor, alcohol dependence, bulimia nervosa and mood disorders [1,2].

We present a case report of an unusual but serious side effect of topiramate. There are a small number of case reports describing topiramate-induced psychosis but the majority are in patients being treated for epilepsy [3-5] and none have described a family history of this side effect.

## Case presentation

A 46-year-old man presented to the emergency medical assessment unit with an 8-day history of visual hallucinations and paranoid delusions. He had a past medical history of essential tremor that was being treated with topiramate. In the past he had tried propranolol and labetalol with little benefit. There was no past history of psychiatric symptoms. He had consumed no alcohol in the previous 3 months but had in the past been drinking eight units of alcohol per day to help control his tremor. No cause for the altered mental state was apparent on clinical examination and initial investigations. The following blood tests were performed and were all normal: full blood count, urea, creatinine, electrolytes, liver function tests, vitamin B12 level, thyroid function tests, autoimmune screen, syphilis serology and blood cultures. On the mini-mental state examination he scored 26/30. A computer tomography scan of the brain and a lumbar puncture were performed and both were normal. There was no evidence of delirium tremens or Wernicke's encephalopathy.

After a discussion with his father on day five of the admission we were told that the onset of the symptoms occurred 2 days after an increase in the dose of topiramate. The patient had commenced topiramate 3 months previously and reached a dose of 75 mg twice a day. The dose had been increased in 25 mg increments. Since the increase in the dose of topiramate and while in the ward he was disorientated as to time and place, had visual and auditory hallucinations and his short-term registration and recall were impaired. He was unable to remember any events occurring after the day the dose of topiramate was increased. The topiramate was stopped and over the following few days he gradually improved. A week after discontinuing the topiramate he was back to his normal self and was discharged. Unfortunately his tremor returned.

After discussing the diagnosis of topiramate-induced psychosis with the patient he told us that his sister had developed visual hallucinations when commenced on topiramate 25 mg twice a day for migraine prophylaxis, and that these had resolved on discontinuing the treatment. She also had no past history of psychiatric symptoms.

## Discussion

Topiramate was originally discovered as an oral hypoglycaemic, later used as an anticonvulsant and is now used as an adjunct to numerous therapies. It is a derivative of monosaccharide D-fructose and the various mechanisms of action include: inhibition of sodium conductance; decreased frequency of generated action potentials; enhanced gamma-aminobutyric acid activity; inhibition of the alpha-amino-3-hydroxy-5-methylisoxazole-4 propionic acid subtype glutamate receptor; and weak inhibition of carbonic anhydrase [1].

The use of topiramate is now well established in the management of epilepsy (both as monotherapy and adjunctive therapy), migraine prophylaxis and essential tremor. There is interest in using it to promote abstinence in alcohol-dependent individuals and in the treatment of bulimia nervosa and mood disorders [1,2]. Common adverse reactions include metabolic acidosis, ataxia, concentration difficulty, confusion, dizziness, fatigue, paresthesiae, somnolence, disturbance of memory, depression, agitation, psychomotor slowness and speech disturbance.

The true prevalence of topiramate-induced psychosis is not well established. Although there have only been a few case reports of topiramate-induced psychosis, the post-marketing anti-epileptic drug survey group found the incidence to be 1.5% in 596 patients. The risk of this side effect may be greater in the general population as studies of topiramate exclude patients with past psychiatric history, and past psychiatric history is the strongest predictor for psychiatric adverse events [6]. However, the risk of developing psychosis in patients with epilepsy is 6 to 12 times higher than in patients without epilepsy, therefore the risk of developing psychosis in patients using topiramate for familial essential tremor may actually be lower than 1.5% (see [6]). There have been several other potential predictors of psychiatric adverse events investigated, such as starting dose and titration schedule [5]. Our patient received the standard dose and titration, starting at 25 mg/day and increased in increments of 25 mg.

Our case report is unique in that a family member (the patient's sister) experienced the same side effect of topiramate-induced psychosis and no other risk factors were identified. Two hypotheses to explain this connection are that there is either an inherited abnormality of topiramate metabolism or a familial predisposition to psychosis. Both have implications for the patient's daughter, who has also been diagnosed with essential tremor at the age of 15.

## Conclusion

Topiramate is being increasingly prescribed Physicians need to be aware of psychosis as a rare but debilitating side effect. Several predictors have been identified and a careful history, including family history, needs to be obtained prior to initiation of therapy to stratify the risk.

## Competing interests

The authors declare that they have no competing interests.

## Consent

Written informed consent was obtained from the patient for publication of this case report and any accompanying images. A copy of the written consent is available for review by the Editor in-Chief of this journal.

## Authors' contributions

RJPJ undertook the literature review and drafting of the manuscript. AC interviewed the patient, contributed to the literature review and revision of the manuscript. CB was responsible for the medical management of the patient and undertook critical review of the manuscript. All authors read and approved the final manuscript.

## References

[B1] Arnone D Review of the use of topiramate for the treatment of psychiatric disorders Ann Gen Psychiatry 2005 4 5 10.1186/1744-859X-4-5 15845141 PMC1088011

[B2] Johnson BA Ait-Daoud N Bowden CL DiClemente CC Roache JD Lawson K Javors MA Ma J Oral topiramate for treatment of alcohol dependence: a randomised controlled trial Lancet 2003 361 1677 1685 10.1016/S0140-6736(03)13370-3 12767733

[B3] Zesiewicz TA Tullidge A Tidwell J Sullivan KL Hauser RA Topiramate-induced psychosis in patients with essential tremor: report of 2 cases Clin Neuropharmacol 2006 29 168 169 10.1097/01.WNF.0000220823.80640.44 16772820

[B4] Khan A Faught E Gilliam F Kuzniecky R Acute psychotic symptoms induced by topiramate Seizure 1999 8 235 237 10.1053/seiz.1999.0287 10452922

[B5] Mula M Trimble MR Lhatoo SD Sander JW Topiramate and psychiatric adverse events in patients with epilepsy Epilepsia 2003 44 659 663 10.1046/j.1528-1157.2003.05402.x 12752464

[B6] Kanner AM Wuu J Faught E Tatum WO 4th Fix A French JA the PADS Investigators A past psychiatric history may be a risk factor for topiramate-related psychiatric and cognitive adverse events Epilepsy Behav 2003 4 548 552 10.1016/j.yebeh.2003.07.007 14527497

